# Statistical Report of Medical and Surgical Cases at the Buffalo Hospital of the Sisters of Charity, from February 22d to September 1, 1849

**Published:** 1849-10

**Authors:** 


*Statistical Report of Medical and Surgical Cases at the Buffalo Hospital of the Sisters of Charity, from February 22d to September* 1, 1849.

The whole number of medical cases received at the Buffalo Hospital of the Sisters of Charity, from February 22 to September 1, 1849, is 337.

The whole number of surgical cases during the same period, 63.

Total of medical and surgical cases, 400.

Of the above number of cases, 134 were cases of Epidemic Cholera, and 33 cases of Cholerine or Diarrhœa tending to cholera. The greater portion of the latter class of cases were brought to the hospital as cases of cholera, but were not registered as such, in consequence of positive evidence of the presence of the diagnostic criteria of that disease not being ascertained. All cases registered as cases of epidemic cholera, were determined by strict observance of rigid principles of diagnosis—not recognizing cholerine as the first stage of cholera.

The number of deaths by epidemic cholera is 52; recoveries 82. The ratio of mortality, therefore, was 1 to a little over $2\frac{1}{2}$ of the whole number of cases.

The number of out patients treated at the hospital, during the same period, is as follows:

| | | | |
|---|---|---|---|
| Medical cases | 13 | | |
| Surgical cases | 19 | | |
| Total | 32 | | |
| The whole number, including in and out patients | | 432 | |
| The number of deaths, exclusive of deaths from epidemic cholera, during the same period, is | | 18 | |
| Do., inclusive of epidemic cholera | | | 70 |

The ratio of mortality, excluding out patients, and deaths from epidemic cholera, is 1 to every $22\frac{2}{9}$ of the whole number received.

Inclusive of deaths by epidemic cholera, the ratio of mortality is 1 to every $5\frac{5}{7}$ of the whole number of patients received.

Excluding all cases of cholera, the ratio of mortality is to every $14\frac{14}{18}$ of the number received.

The following table exhibits the number of cases of the various diseases, respectively, occurring during the same period, with the results of the cases of each disease:

| Diseases. | Whole No. | Recov: | Died. | Improv'd. | Not Im. |
|---|---|---|---|---|---|
| Pulmo-tuberculosis | 15 | . | 5 | 5 | 5 |
| Intermittent Fever | 26 | 26 | . | . | . |
| Epidemic Cholera | 134 | 82 | 52 | . | . |
| Diarrhœa | 33 | 28 | 3 | 2 | . |
| Dysentery | 9 | 7 | 2 | . | . |
| Common Continued Fever | 9 | 9 | . | . | . |
| Typhus Fever | 10 | 7 | 3 | . | . |
| Remittent Fever | 4 | 4 | . | . | . |
| Erysipelas | 3 | 3 | . | . | . |
| Varioloid—all sent to small-pox hospital | 3 | . | . | . | . |
| Eczema | 1 | . | . | . | . |
| Erythema Nodosum | 1 | 1 | . | . | . |
| Acne Indurata | 1 | 1 | . | . | . |
| Tonsillitis | 1 | 1 | . | . | . |
| Pharyngitis | 1 | 1 | . | . | . |
| Pneumonitis | 2 | 2 | . | . | . |
| Pleuritis | 1 | 1 | . | . | . |
| Pertussis | 1 | 1 | . | . | . |
| Prurigo | 1 | 1 | . | . | . |
| Hypochondriasis | 1 | 1 | . | . | . |
| Neuralgia | 4 | 4 | . | . | . |
| Pleurodynia | 4 | 4 | . | . | . |
| Rheumatism | 1 | 1 | . | . | . |
| Anæmic Coma | 1 | . | 1 | . | . |

| Diseases. | Whole No. | Rec'ov. | Died. | Improv'd. | Not Im. |
|---|---|---|---|---|---|
| Menorrhagia | 2 | 2 | . | . | . |
| Cholera Morbus | 3 | 3 | . | . | . |
| Epilepsy | 1 | . | . | . | 1 |
| Hemiplegia | 1 | . | 1 | . | . |
| Salivation | 3 | 3 | . | . | . |
| Colic | 2 | 2 | . | . | . |
| Colica Pictonum | 1 | 1 | . | . | . |
| Cerebro-Spinal Meningitis | 1 | . | 1 | . | . |
| Delirium Tremens } In the two fatal cases, the disease followed epidemic cholera. | 3 | 1 | 2 | . | . |
| | 297 | 197 | 70 | 11 | 2 |

Diseases not particularly stated, and patients now remaining at hospital, 40.

The following is a list of the surgical diseases during the same period, with the number of cases of each disease:

*Fracture of* femur, number of cases, 4; tibia und fibula, 1; fibula, with contused and lacerated wound, 1; radius and ulna, 2; humerus, 1; base of scull and injury of spinal cord, 1; necrosis of humerus, 1; necrosis of alveoli, 1; sacral abscess and caries, 1; conjunctivitis, 7; ophthalmia, 3; amaurosis, 2; nebulæ and contracted pupil, 1; granular conjunctivitis, 1; otitis, 2; syphilis—primary, secondary, and tertiary, 7; hæmorrhoids, 1; torticollis, and operation, 1; ulcers, 4; burns, 2; gonorrhœa, 1; incised wounds, 2; contused do., 2; punctured do., 1; gunshot do., 1; contusions, 4; sprained ancle, 2; do. shoulder, 1; furunculus, 1; pectoral abscess, 1; inflammation of palm of hand, 1; scrofulosis and abscess, 1; total, 63.

The following is a list of surgical diseases of out patients for the same period:

Varicose veins and ulcers; contusion of scull; scrofula; fracture of olecranon; fracture of clavicle; ophthalmia; fracture of finger, 1st phalanx; otitis; burn of leg; gonorrhœa; pterygium; conjunctivitis; fracture of radius and ulna; laceration of integumen ; phlegmonous inflammation of leg; abscess of head; lacerated wound—bitten by a dog; amaurosis; syphilis; total, 19.

By comparison with the first semi-annual report of this Institution, contained in the April number of this Journal, 1849, it will be perceived that the number of cases received for the last half year is more than trebled. Excluding cases of epidemic cholera, the number of cases is more than doubled. When it is considered that this is the only hospital at present in this city, and that the majority of the cases relieved, if this Institution did not exist, would, necessarily, have come under the provisions for pauper relief, some idea may be formed of the nature and extent of the charitable aid it has already afforded the indigent sick, especially when the ratio of mortality at the poor-house of this county is taken into account. [See number of this Journal for Dec. 1848]. It is only to be regretted that its benefits could not have been still farther extended — but of this more anon.

The new addition, when completed, will enable the directors of the hospital to extend relief to twice as many as the present building will accommodate.

As a field for clinical illustrations of medical and surgical diseases, it affords a number and variety of cases sufficiently ample for all the advantages to be derived, by the medical student, from this province of medical instruction.

